# Changes in Neutrophil–Lymphocyte or Platelet–Lymphocyte Ratios and Their Associations with Clinical Outcomes in Idiopathic Pulmonary Fibrosis

**DOI:** 10.3390/jcm10071427

**Published:** 2021-04-01

**Authors:** Steven D. Nathan, Jayesh Mehta, John Stauffer, Elizabeth Morgenthien, Ming Yang, Susan L. Limb, Sangeeta Bhorade

**Affiliations:** 1 Advanced Lung Disease and Transplant Program, Inova Fairfax Hospital, 3300 Gallows Rd, Falls Church, VA 22042, USA; 2 Feinberg School of Medicine, Northwestern University, 420 E Superior St, Chicago, IL 60611, USA; jmehta@nm.org (J.M.); sangeeta@veracyte.com (S.B.); 3 Genentech, Inc., 1 DNA Way, South San Francisco, CA 94080, USA; stauffer.john@gene.com (J.S.); morgente@gene.com (E.M.); yang.ming.ym1@gene.com (M.Y.); limb.susan@gene.com (S.L.L.)

**Keywords:** NLR, PLR, idiopathic pulmonary fibrosis, clinical outcomes, biomarker

## Abstract

Identification of prognostic and predictive biomarkers in idiopathic pulmonary fibrosis (IPF) could aid assessment of disease severity and prediction of progression and response to treatment. This analysis examined reference ranges for neutrophil–lymphocyte ratio (NLR) and platelet–lymphocyte ratio (PLR) in IPF, and the relationship between NLR or PLR changes and clinical outcomes over 12 months. This post hoc analysis included patients with IPF from the Phase III, double-blind trials of pirfenidone, ASCEND (NCT01366209) and CAPACITY (NCT00287716 and NCT00287729). The relationship between change from baseline to Month 12 in NLR or PLR (divided into quartiles (Q1–Q4)) and outcomes (mortality, respiratory hospitalization, declines in lung function, exercise capacity and quality of life) was assessed. Estimated reference ranges at baseline for all patients analyzed (*n* = 1334) were 1.1–6.4 for NLR and 56.8–250.5 for PLR. Significant trends were observed across NLR and PLR quartiles for all outcomes in placebo-treated patients, with patients manifesting the greatest NLR or PLR changes experiencing the worst outcomes. These results suggest that the greatest NLR or PLR changes over 12 months were associated with worse clinical outcomes. Further research is needed to determine the utility of NLR and PLR as prognostic biomarkers in IPF.

## 1. Introduction

Idiopathic pulmonary fibrosis (IPF) is a progressive lung disease of unknown etiology that is associated with poor prognosis [1]. Patients with IPF usually experience a progressive worsening of exertional dyspnea and reductions in lung function [1]. Antifibrotic treatment with pirfenidone or nintedanib can slow IPF progression [2,3,4].

Identification of prognostic and predictive biomarkers could have multiple uses in IPF, including the assessment of risk and disease severity, the prediction of progression and response to treatment, and the measurement of treatment responses [5,6]. The neutrophil–lymphocyte ratio (NLR) and platelet–lymphocyte ratio (PLR) have been used as markers of inflammation, with higher values found to be associated with worse outcomes in multiple diseases, including rheumatoid arthritis, pancreatitis, cardiovascular diseases and various cancers [7,8,9,10,11]. NLR and/or PLR have also been evaluated as prognostic markers in chronic obstructive pulmonary disease [12] and IPF [13]. 

The scope of this analysis was to explore the association between NLR or PLR and clinical outcomes in IPF in order to pave the way for further research into the value of NLR and PLR as potential prognostic or predictive biomarkers in IPF. We investigated reference ranges for NLR and PLR in IPF and the relationship between NLR or PLR and clinical outcomes over 12 months in patients with IPF using data from the ASCEND [2] and CAPACITY [3] studies of pirfenidone and the GIPF-001 and GIPF-007 studies of interferon gamma-1b (IFN-γ-1b) [14,15]. Some of the results of this analysis have been previously reported in the form of an abstract [16].

## 2. Methods

### 2.1. Patient Population

This post hoc analysis included patients randomized to receive treatment with pirfenidone or placebo in the Phase III, double-blind trials, ASCEND (Study 016; NCT01366209) and CAPACITY (Studies 004 and 006; NCT00287716 and NCT00287729) [2,3]. Patients enrolled in ASCEND and CAPACITY were between 40 and 80 years of age, with a confirmed diagnosis of IPF using high-resolution computed tomography alone or in combination with a surgical lung biopsy [2,3]. 

Patients with IPF from two independent Phase III, double-blind, placebo-controlled trials of IFN-γ-1b (GIPF-001 (NCT00047645) and GIPF-007 (NCT00075998)) were analyzed and used as a validation cohort [14,15]. Although neither trial showed a beneficial effect of IFN-γ on pulmonary function, progression-free survival, quality of life or mortality [14,15], only patients in the placebo arm were included in this analysis since IFN-γ is known to affect hematopoiesis, which could have impacted the cell types constituting the ratios [17]. 

ASCEND, CAPACITY, GIPF-001 and GIPF-007 were conducted in accordance with the International Conference on Harmonisation guidelines and the Declaration of Helsinki, and the relevant local legal and regulatory requirements of the countries in which the trials were conducted. All patients provided written informed consent. 

### 2.2. Reference Ranges

NLR was calculated as the absolute neutrophil count divided by the absolute lymphocyte count, and PLR was calculated as the absolute platelet count divided by the absolute lymphocyte count. NLR and PLR were assessed at baseline in patients pooled from the pirfenidone (2403 mg/day and 1197 mg/day) and placebo groups of ASCEND and CAPACITY to calculate reference ranges.

Reference ranges were developed using a distribution-free method. For each ratio, upper and lower limits of the reference range were estimated as the data (or interpolated) values corresponding to the 97.5th and 2.5th percentiles, respectively. Confidence intervals for the upper and lower limits were derived from order statistics that most closely provided 90% confidence for the two population percentiles. To evaluate the appropriateness of reporting a single reference range for all patients, separate estimates (confidence intervals) were obtained for each sex (male, female) and age group (<65 years, ≥65 years), and comparisons between demographic groups were based on two-sample *t*-tests applied to log-transformed values.

### 2.3. Baseline NLR and PLR

For each treatment arm (pirfenidone 2403 mg/day and placebo), patients were ranked by baseline NLR or PLR and divided into quartiles (Q1–Q4); summary statistics were obtained for each quartile. The relationship between baseline NLR or PLR quartile and clinical outcomes (all-cause mortality, absolute decline in percent predicted forced vital capacity (FVC) ≥10% or death, absolute decline in 6-min walking distance (6MWD) ≥50 m or death, a worsening in University of California San Diego Shortness of Breath Questionnaire (UCSD-SOBQ) score ≥20 points or death, any respiratory hospitalization, and any respiratory hospitalization or death) at Month 12 was assessed separately in patients from the pirfenidone 2403 mg/day and placebo groups of ASCEND and CAPACITY (the pirfenidone 1197 mg/day group was excluded from trend analyses because pirfenidone at the daily dose of 1197 mg is known to be less effective than 2403 mg) [3]. Absolute decline in percent predicted diffusing capacity for carbon monoxide (DLco) ≥15% or death was also an endpoint; however, fewer data points were available for this endpoint because post-baseline percent predicted DLco was only recorded in CAPACITY. The quartiles were not redefined for this subset.

The Cochran–Armitage test (exact version) was used to assess trends across the quartiles, using quartile integers (1, 2, 3 and 4) as scores. Sensitivity analyses were conducted using median changes as scores for the quartiles.

### 2.4. Changes in NLR and PLR

NLR and PLR changes were calculated as the last ratio obtained on or before Month 12 minus the baseline ratio. The percentage change from baseline at Month 12 was calculated for the neutrophil and lymphocyte count (for NLR) and the platelet and lymphocyte count (for PLR) to assess which cell type drives change in the ratios. For patients who died or discontinued prior to Month 12, the last available post-baseline value was used to calculate the ratio.

For each ratio and treatment arm (pirfenidone 2403 mg/day and placebo), patients were ranked by ratio changes over 12 months and divided into quartiles (Q1–Q4); summary statistics were obtained for each quartile. The relationship between changes in NLR or PLR from baseline to Month 12 and clinical outcomes at Month 12 was assessed separately in patients from the pirfenidone 2403 mg/day and placebo groups of ASCEND and CAPACITY. The Cochran–Armitage test and sensitivity analyses were conducted as described previously. Kaplan–Meier plots were constructed to examine time to death from any cause in the placebo group only, comparing Q1–Q3 versus Q4 for the change from baseline to Month 12 in NLR and PLR using the log-rank test. Q1–Q3 were pooled due to the small number of events occurring in these quartiles.

### 2.5. Validation Using GIPF-001 and GIPF-007 Cohorts

The placebo groups from GIPF-001 and GIPF-007 were used to validate the findings for all-cause mortality. NLR and PLR and changes from baseline to Month 12 were calculated as described previously. Patients from both trials combined were assigned to quartiles based on the changes in each ratio, and the Cochran–Armitage test (exact version) was used to assess trends in all-cause mortality.

## 3. Results

### 3.1. Patients

Overall, 1334 patients were randomized in ASCEND and CAPACITY (pirfenidone, *n* = 710; placebo, *n* = 624). In these patients, the median (Q1, Q3) NLR and PLR values were 2.5 (1.8, 3.3) and 119.3 (93.7, 150.3), respectively. Patient demographics and baseline characteristics have been previously described [2,3] and did not differ considerably in patients categorized by baseline NLR or PLR, or by change in NLR or PLR (data categorized by NLR are shown in Table 1 and Supplementary Materials Tables S1–S3).

### 3.2. Estimated Reference Ranges

Reference ranges were calculated from baseline values of all patients randomized in ASCEND and CAPACITY. At baseline, estimated reference ranges for NLR and PLR were 1.1–6.4 and 56.8–250.5, respectively (Table 2). For NLR, statistically significant differences were observed between sexes and age groups (*p* = 0.001); although lower limits of the reference ranges were similar, upper limits were lower for females than for males, and for patients <65 versus ≥65 years of age. For PLR, a statistically significant difference was observed between sexes (*p* = 0.03), but not between age groups (*p* = 0.17); however, estimated reference range limits were generally similar between groups (<10% difference; Table 2).

### 3.3. Baseline NLR and PLR

In the placebo group, median baseline values for Q1, Q2, Q3 and Q4 were 1.5, 2.2, 2.9 and 4.1, respectively, for NLR, and 79.2, 105.2, 136.9 and 180.3, respectively, for PLR. Significant trends for all-cause mortality and absolute decline in 6MWD ≥50 m or death were observed across baseline NLR quartiles; a significant trend for absolute decline in 6MWD ≥50 m or death was also observed across baseline PLR quartiles (Supplementary Materials Table S4).

In the pirfenidone 2403 mg/day group, median baseline values for Q1, Q2, Q3 and Q4 were 1.5, 2.1, 2.8 and 4.0, respectively, for NLR, and 78.7, 105.3, 131.9 and 181.9, respectively, for PLR. Significant trends were observed across the baseline NLR quartiles for absolute decline in percent predicted FVC ≥10% or death, absolute decline in 6MWD ≥50 m or death, and a worsening in UCSD-SOBQ score ≥20 points or death (Supplementary Materials Table S4).

There was no significant relationship between baseline NLR or PLR and the other clinical outcomes assessed (Supplementary Materials Table S4).

### 3.4. Changes in NLR

In the placebo group, the median 12-month change in NLR by quartile was −0.8, −0.1, 0.4 and 1.6 for Q1, Q2, Q3 and Q4 (as defined by the change from baseline to Month 12 in NLR), respectively (Table 3). Significant trends were observed across the NLR change quartiles for all outcomes, with patients with the greatest change in NLR (Q4) experiencing the worst outcomes. Similar declines in percent predicted FVC from baseline to Month 12 were observed in the Q1, Q2 and Q3 cohorts compared with a slightly greater decline in percent predicted FVC in the Q4 cohort. Differences across quartiles in NLR changes were driven by increased neutrophil count and decreased lymphocyte count between baseline and Month 12 from Q1 to Q4 (Table 3). 

**Figure 1:**
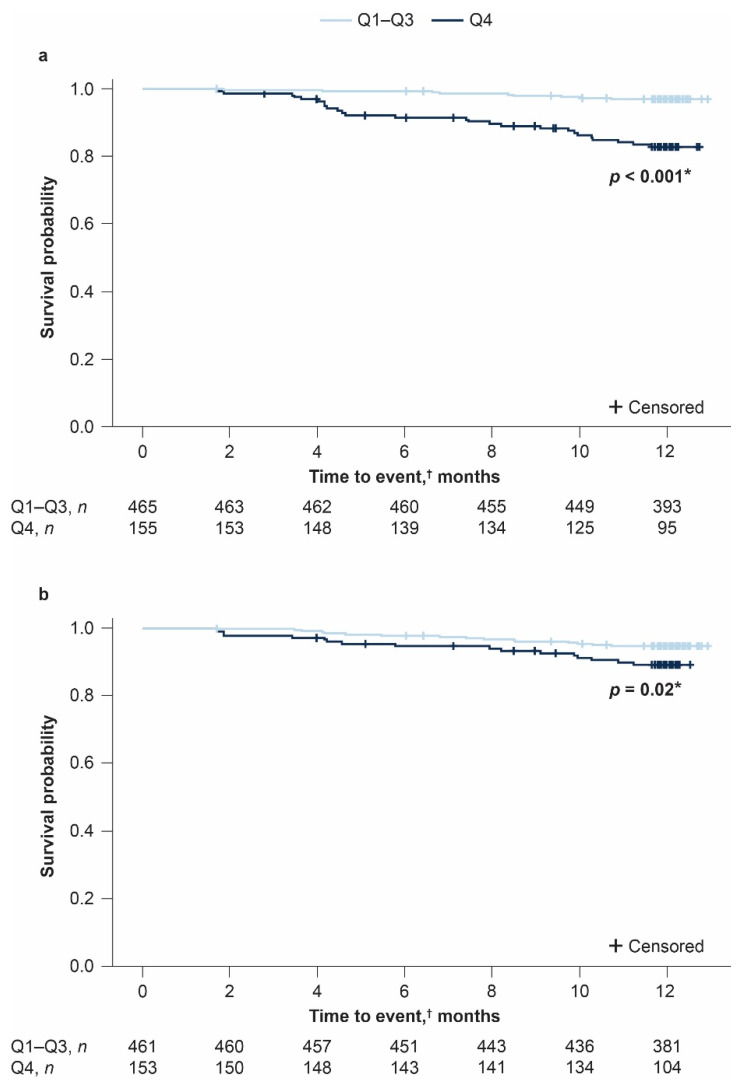
Kaplan–Meier plots of all-cause mortality based on quartiles as defined by (**a**) neutrophil–lymphocyte ratio (NLR) and (**b**) platelet–lymphocyte ratio (PLR) changes from baseline to Month 12 in patients with idiopathic pulmonary fibrosis (IPF) (pooled from the placebo groups of ASCEND and CAPACITY). * Log-rank test *p*-value. ^†^ Six patients had their Month 12 assessment at or after 12.5 months. Q: quartile.

All-cause mortality in patients with the greatest NLR changes (Q4) was significantly higher (*p* < 0.001) than in patients in the combined Q1–Q3 cohort (median (Q1, Q3) change in NLR: 1.6 (1.1, 2.7) vs. −0.1 (−0.6, 0.3), respectively). Specifically, over the 12-month period, 15/465 (3.2%) patients in the combined Q1–Q3 cohort and 26/155 (16.8%) patients in the Q4 cohort died. Survival at 12 months based on all-cause mortality was 96.8% among patients in the combined Q1–Q3 cohort compared with 82.7% among patients in the Q4 cohort (Figure 1a).

In the pirfenidone 2403 mg/day group, the median 12-month change in NLR by quartile was −0.6, 0.1, 0.7 and 1.8 for Q1, Q2, Q3 and Q4, respectively. Significant trends were observed across quartiles for the absolute decline in percent predicted FVC ≥10% or death, any respiratory hospitalization, and any respiratory hospitalization or death, but not for the other clinical outcomes assessed (Supplementary Materials Table S4).

### 3.5. Changes in PLR

In the placebo group, the median 12-month change in PLR by quartile was −35.7, −6.1, 12.2 and 49.0 for Q1, Q2, Q3 and Q4 (as defined by the change from baseline to Month 12 in PLR), respectively (Supplementary Materials Table S5). Significant trends were observed across the PLR change quartiles for all outcomes, with patients with the greatest change in PLR (Q4) experiencing the worst outcomes. Similar declines in percent predicted FVC from baseline to Month 12 were observed in the Q1, Q2 and Q3 cohorts, compared with a slightly greater decline in percent predicted FVC in the Q4 cohort. Differences across quartiles in PLR changes appeared to be driven more by decreased lymphocyte count than increased platelet count between baseline and Month 12 from Q1 to Q4 (Supplementary Materials Table S5). 

All-cause mortality in patients with the greatest PLR changes (Q4) was significantly higher (*p* = 0.02) than in patients in the combined Q1–Q3 cohort (median (Q1, Q3) change in PLR: 49 (37.1, 70.5) vs. −6.1 (−24.4, 7.6), respectively). Specifically, over the 12-month period, 24/461 (5.2%) patients in the combined Q1–Q3 cohort and 16/153 (10.5%) patients in the Q4 cohort died. Survival at 12 months based on all-cause mortality was 94.8% among patients in the combined Q1–Q3 cohort compared with 89.3% among patients in the Q4 cohort (Figure 1b).

In the pirfenidone 2403 mg/day group, the median 12-month change in PLR by quartile was −25.1, 3.9, 25.9 and 73.7 for Q1, Q2, Q3 and Q4, respectively. No significant trends were observed across the PLR quartiles for any of the clinical outcomes (Supplementary Materials Table S4).

### 3.6. Validation Using the GIPF-001 and GIPF-007 Cohort

The validation cohort of patients, which included patients from the placebo groups of GIPF-001 and GIPF-007, comprised 437 patients with NLR measurements and 431 patients with PLR measurements. To be included in this analysis, patients were required to have both a baseline and at least one post-baseline ratio value available at or before Month 12.

The median 12-month change in NLR was −1.8, −0.2, 0.5 and 2.8 for Q1, Q2, Q3 and Q4 (as defined by the change from baseline to Month 12 in NLR), respectively (Table 4). Over the 12-month period, 8/110 (7.3%), 3/109 (2.8%), 6/109 (5.5%) and 21/109 (19.3%) patients died in the Q1, Q2, Q3 and Q4 cohorts, respectively (*p* = 0.001; Table 4).

The median 12-month change in PLR was −53.3, −10.6, 14.9 and 81.4 for Q1, Q2, Q3 and Q4 (as defined by the change from baseline to Month 12 in PLR), respectively (Table 4). Over the 12-month period, 9/108 (8.3%), 2/108 (1.9%), 9/108 (8.3%) and 18/107 (16.8%) patients died in the Q1, Q2, Q3 and Q4 cohorts, respectively (*p* = 0.01; Table 4).

## 4. Discussion

The results of this post hoc analysis suggest that patients with IPF with the greatest change in NLR or PLR over 12 months may be at the highest risk of poor outcomes, including mortality, respiratory hospitalization, and declines in lung function, 6MWD and quality of life. The change in NLR was driven by both increased neutrophil and decreased lymphocyte counts, whereas PLR change appeared to be driven more by decreased lymphocyte count than increased platelet count. This suggests that change in NLR, in particular, could have potential as a non-invasive prognostic biomarker in IPF, given the lack of additional information provided by the change in PLR. Unlike gene or protein signatures, blood cell counts are already a routine part of clinical monitoring and would therefore be easier to adopt in clinical practice without the need for additional testing or incremental cost.

Although significant associations were found between baseline NLR and certain clinical outcomes, these associations were not consistent across all outcomes, and baseline PLR did not show significant associations with most outcomes. Furthermore, although significant associations for NLR or PLR change were observed consistently for all outcomes for patients treated with placebo, this was not the case for patients treated with pirfenidone 2403 mg/day. These findings indicate that NLR or PLR change could potentially be a more robust prognostic biomarker than baseline NLR or PLR alone, but may be less suitable as a predictive biomarker for patients receiving treatment. The relationships between NLR or PLR change and clinical outcomes were observed in both the Cochran–Armitage trend tests and sensitivity analyses (using quartile integers and the observed median change as scores, respectively), indicating that choice of scoring did not impact the trends observed.

Other blood biomarkers are being investigated in IPF, although, to our knowledge, this is the first time that NLR and PLR changes have been investigated together for their association with IPF outcomes. A previous study that assessed baseline NLR in two cohorts of patients with IPF found that mortality was higher in patients with high baseline NLR versus low baseline NLR, independent of GAP (Gender, Age, Physiology) score [13]. Another study that assessed NLR using bronchoalveolar lavage (BAL) samples from 59 patients with IPF found that NLR was inversely correlated with FVC and forced expiratory volume in 1 s, and positively correlated with composite physiologic index, measured at the same time as collection of the BAL sample [18].

In a further study, the association between the mortality and baseline levels of white blood cells (WBCs), including polymorphonuclear (PMN) cells (neutrophils, eosinophils, basophils and mast cells) was investigated in two independent cohorts of patients with IPF from the United States and Turkey [19]. This study found that WBC and PMN counts were associated with increased risk of death or lung transplantation. Additionally, there was an association between PMN/lymphocyte ratio and outcomes, which was likely driven primarily by PMN cell count [19]. This is similar to the findings of our current analysis, where NLR change was not only associated with mortality but also with other outcomes indicative of disease progression in IPF. In addition, our analysis found that the NLR change was driven by changes in both neutrophil and lymphocyte counts.

Another study analyzed peripheral blood mononuclear cell samples using multiple cohorts of patients with IPF and other fibrotic diseases, and found that baseline monocyte and neutrophil counts, but not lymphocyte or eosinophil counts, were associated with survival in two IPF cohorts [20]. Neutrophil count was also associated with mortality in propensity-matched non-fibrotic cohorts. The authors suggested that the neutrophil count could be a marker of poor prognosis for fibrotic lung disease in general, whereas monocyte count appeared to be more specific for IPF prognosis [20].

These findings showing associations between certain WBC counts and IPF outcomes suggest that a systemic inflammatory state exists in patients with IPF. Neutrophils, monocytes and lymphocytes have all been linked with the pathogenesis of IPF; changes in these blood cells have been found both in the lungs (neutrophils and lymphocytes) [21,22,23,24] and circulation (monocytes and lymphocytes) [22,23,24,25]. IPF is thought to result from recurrent, subclinical epithelial damage leading to the aberrant repair of injured alveoli [1], so there is likely to be a systemic inflammatory response associated with this tissue injury [19]. However, the relationship between local and systemic inflammation may not always be clear, and the mechanism(s) by which systemic inflammation may arise and how this might reflect disease progression in patients with IPF are not yet well understood.

A proposed mechanism that might contribute to this possible systemic inflammatory response involves necroptosis of alveolar epithelial cells; necroptosis is a type of programmed cell death that can trigger innate and adaptive immune responses through the release of damage-associated molecular patterns [26]. Additionally, autoimmunity has been implicated in the pathogenesis of IPF, which could also contribute to an inflammatory state [19,27]. Moreover, changes in the lung microbiome are thought to influence IPF perpetuation and outcomes, and might be associated with a systemic inflammatory response [19,28]. Nevertheless, it is possible that the systemic inflammatory response is not directly related to IPF but is a result of other factors, such as subclinical infection or underlying comorbid conditions [19]. 

Our analysis also aimed to establish reference ranges for NLR and PLR in patients with IPF. The upper limits of estimated reference ranges for NLR and PLR at baseline in our analysis (6.4 and 250.5, respectively) were slightly higher than the reported upper limits of reference ranges among a variety of healthy adult populations (2.2–4.4 and 199–218, respectively) [7,29,30]. Consistent with these observations, higher reference ranges for both NLR and PLR have been reported for various conditions, including rheumatoid arthritis [11], cardiovascular disease [9,31] and pancreatic cancer [8], compared with healthy patients.

There are a number of limitations that should be considered when interpreting these findings, including the post hoc nature of this analysis of pooled data from Phase III studies, which prevents adequate control of type I errors, and the lack of stratification of randomization by quartile. Additionally, if NLR and PLR are indeed biomarkers of the risk of progression, then it is likely that the trial populations had a lower median NLR and PLR at baseline, and smaller changes over 12 months, than might be expected in a real-world population of patients, due to the exclusion of patients with greater lung-function impairment or certain comorbidities from the trials. Furthermore, these analyses were univariate and thus did not account for potential confounding variables; nevertheless, the mortality findings were supported by the results in the validation cohort. The univariate nature of these analyses also meant that we could not determine whether baseline factors such as pulmonary function, exercise capacity or GAP score were associated with NLR or PLR. Moreover, NLR or PLR changes were only calculated at Month 12, which means that the results do not confirm whether NLR or PLR changes are prognostic or predictive of any particular outcomes, because clinical outcomes may occur earlier than this (with the exception of mortality, since the last observation was carried forward in this case), and we cannot rule out the possibility that changes in NLR or PLR are influenced by infections or acute exacerbations. However, these were intended to be preliminary analyses to explore the associations between NLR or PLR and IPF outcomes in order to provide a foundation for future research into the potential of NLR and PLR as prognostic or predictive biomarkers in IPF. 

In conclusion, these results suggest that patients with the greatest increase in NLR or PLR over 12 months had poorer outcomes, including mortality, respiratory hospitalization, and decline in lung function, 6MWD and quality of life, compared with patients who experienced smaller changes in these readily available ratios. The changes in both neutrophil and lymphocyte counts in relation to each other across the NLR quartiles were more pronounced and consistent than the corresponding changes across the PLR quartiles, which largely reflected changes in lymphocyte counts only, suggesting that NLR could be a better ratio to monitor than PLR. Further research is needed to investigate the potential value of NLR and PLR as prognostic or predictive biomarkers for outcomes among patients with IPF.

## Figures and Tables

**Table 1:** Baseline demographics and clinical characteristics of patients included in ASCEND and CAPACITY (placebo and pirfenidone 2403 mg/day groups), by change from baseline to Month 12 in NLR.

	Placebo				Pirfenidone 2403 mg/Day			
	Q1 (*n* = 155)	Q2 (*n* = 155)	Q3 (*n* = 155)	Q4 (*n* = 155)	Q1 (*n* = 155)	Q2 (*n* = 155)	Q3 (*n* = 155)	Q4 (*n* = 154)
Male sex, *n* (%)	115 (74.2)	118 (76.1)	120 (77.4)	108 (69.7)	117 (75.5)	113 (72.9)	116 (74.8)	115 (74.7)
Age, year, mean (SD)	67.5 (7.4)	66.6 (7.5)	65.9 (8.1)	68.5 (7.0)	66.4 (8.2)	66.7 (6.9)	66.8 (7.8)	69.0 (7.2)
Percent predicted FVC, mean (SD)	71.6 (13.4)	74.3 (14.3)	70.4 (12.2)	71.6 (14.3)	73.0 (13.1)	72.1 (13.3)	70.7 (13.9)	70.9 (12.4)
Percent predicted DLco, mean (SD)	45.3 (9.6)	46.1 (9.7)	46.0 (10.1)	44.9 (14.4) *	46.6 (9.9)	45.9 (9.6)	45.3 (10.8)	44.7 (10.4)
Hemoglobin count, g/L, median (Q1, Q3)	140.0 (129.0, 149.0)	143.0 (135.0, 151.0)	143.0 (136.0, 153.0)	140.0 (131.0, 150.0)	142.0 (132.0, 152.0)	143.0 (135.0, 151.0)	142.0 (133.0, 151.0)	142.5 (135.0, 149.0)
Hematocrit count, median (Q1, Q3)	0.42 (0.39, 0.45)	0.43 (0.41, 0.45)	0.42 (0.40, 0.46)	0.42 (0.39, 0.45)	0.42 (0.40, 0.45)	0.43 (0.40, 0.45)	0.43 (0.40, 0.45)	0.42 (0.40, 0.45)
Platelet count, GI/L, median (Q1, Q3)	240.0 (206.0, 296.0)	240.0 (199.0, 280.0)	240.0 (199.0, 287.0)	239.0 (207.0, 278.0)	243.0 (214.0, 291.0)	229.0 (196.0, 275.0)	240.0 (206.0, 278.0)	236.0 (196.0, 281.0)
White blood cell count, GI/L, median (Q1, Q3)	8.5 (7.3, 10.1)	7.7 (6.6, 8.9)	7.8 (6.8, 8.8)	7.9 (6.5, 8.9)	8.7 (7.2, 9.7)	7.7 (6.4, 8.8)	7.6 (6.7, 8.9)	7.8 (6.5, 8.8)
Neutrophil count, GI/L, median (Q1, Q3)	5.8 (4.9, 7.3)	4.8 (4.0, 5.6)	4.6 (3.9, 5.7)	4.8 (4.0, 5.8)	5.7 (4.8, 7.0)	4.6 (3.7, 5.5)	4.6 (3.9, 5.6)	4.8 (3.9, 5.8)
Lymphocyte count, GI/L, median (Q1, Q3)	1.8 (1.5, 2.1)	2.1 (1.7, 2.6)	2.2 (1.9, 2.8)	2.0 (1.6, 2.5)	2.0 (1.4, 2.3)	2.1 (1.7, 2.7)	2.1 (1.8, 2.7)	2.0 (1.5, 2.4)
Monocyte count, GI/L, median (Q1, Q3)	0.46 (0.36, 0.60)	0.46 (0.35, 0.55)	0.48 (0.39, 0.60)	0.47 (0.38, 0.57)	0.48 (0.39, 0.60)	0.47 (0.40, 0.58)	0.48 (0.38, 0.58)	0.47 (0.39, 0.56)
Eosinophil count, GI/L, median (Q1, Q3)	0.20 (0.13, 0.30)	0.20 (0.13, 0.30)	0.23 (0.14, 0.33)	0.25 (0.17, 0.36)	0.20 (0.12, 0.33)	0.21 (0.14, 0.32)	0.24 (0.14, 0.33)	0.24 (0.14, 0.37)
Basophil count, GI/L, median (Q1, Q3)	0.05 (0.04, 0.07)	0.05 (0.03, 0.07)	0.05 (0.04, 0.07)	0.05 (0.04, 0.07)	0.05 (0.04, 0.07)	0.05 (0.04, 0.07)	0.05 (0.04, 0.07)	0.05 (0.04, 0.06)
NLR, median (Q1, Q3)	3.2 (2.7, 4.3)	2.3 (1.7, 3.2)	2.0 (1.5, 2.6)	2.4 (1.9, 3.2)	3.0 (2.3, 4.1)	2.2 (1.7, 2.7)	2.3 (1.6, 2.8)	2.5 (1.8, 3.3)
PLR, median (Q1, Q3)	138.5 (110.0, 172.4) *	114.2 (91.7, 148.0)	105.6 (82.6, 140.4) ^†^	117.5 (97.0, 152.3) ^‡^	129.5 (104.7, 171.8)	106.0 (86.6, 139.2) ^‡^	117.6 (89.4, 141.9)	123.1 (95.8, 150.0)

* *n* = 153. ^†^ *n* = 154. ^‡^ *n* = 152. DLco: diffusing capacity for carbon monoxide; FVC: forced vital capacity; GI: 10^9^ cells; NLR: neutrophil–lymphocyte ratio; PLR: platelet–lymphocyte ratio; Q: quartile; SD: standard deviation.

**Table 2:** NLR and PLR reference ranges in patients with IPF (pooled from all treatment groups in ASCEND and CAPACITY).

	Patient Group	Number of Values	Lower Limit (2.5th Percentile)	Upper Limit (97.5th Percentile)
Estimated Reference Ranges (90% CI)				
NLR	All patients	1334	1.06 (1.01–1.09)	6.38 (5.90–6.98)
	Male	993	1.10 (1.05–1.16)	6.60 (5.95–7.27)
	Female	341	0.96 (0.89–1.06)	4.99 (4.54–6.98)
	<65 year	468	1.08 (0.99–1.15)	5.90 (5.06–6.91)
	≥65 year	866	1.05 (0.96–1.10)	6.60 (5.92–7.27)
PLR	All patients	1323	56.79 (54.55–57.75)	250.45 (240.52–275.46)
	Male	986	56.41 (54.55–57.75)	252.13 (238.20–279.82)
	Female	337	56.97 (46.42–64.09)	248.60 (240.00–303.03)
	<65 year	466	55.49 (46.79–61.01)	240.00 (229.09–258.09)
	≥65 year	857	56.97 (52.52–59.31)	263.11 (246.88–289.83)

CI: confidence interval; IPF: idiopathic pulmonary fibrosis; NLR: neutrophil–lymphocyte ratio; PLR: platelet–lymphocyte ratio.

**Table 3:** Month 12 endpoints based on quartiles as defined by NLR changes from baseline to Month 12 in patients with IPF (pooled from the placebo groups of ASCEND and CAPACITY).

	Q1 (*n* = 155)	Q2 (*n* = 155)	Q3 (*n* = 155)	Q4 (*n* = 155)	Cochran–Armitage *p*-Value *
NLR change from baseline ^†^ to Month 12, ^‡^ median (Q1, Q3)	−0.8 (−1.4, −0.6)	−0.1 (−0.2, 0.0)	0.4 (0.3, 0.6)	1.6 (1.1, 2.7)	–
Neutrophils percent change from baseline to Month 12, median (Q1, Q3)	−18.5 (−30.9, −8.1)	−1.5 (−12.3, 10.9)	18.2 (1.3, 31.0)	34.9 (19.9, 74.3)	–
Lymphocytes percent change from baseline to Month 12, median (Q1, Q3)	16.8 (1.9, 36.1)	3.2 (−8.8, 14.9)	−5.3 (−16.6, 8.6)	−17.4 (−32.2, −8.2)	–
Absolute decline in percent predicted FVC from baseline to Month 12, median (Q1, Q3)	−4.4 (−8.6, −1.8)	−3.7 (−8.6, 0.1)	−4.5 (−8.9, −1.1)	−9.4 (−18.1, −2.9)	–
All-cause mortality, *n* (%)	3 (1. 9)	5 (3.2)	7 (4.5)	26 (16.8)	<0.001
Absolute decline in percent predicted FVC ≥10% or death, *n* (%)	26 (16.8)	29 (18.7)	35 (22.6)	73 (47.1)	<0.001
Absolute decline in 6MWD ≥50 m or death, *n* (%)	48 (31.0)	46 (29.7)	44 (28.4)	72 (46.5)	0.009
Worsening in UCSD-SOBQ score ≥20 points or death, *n* (%)	34 (21.9)	38 (24.5)	42 (27.1)	78 (50.3)	<0.001
Any respiratory hospitalization, *n* (%)	9 (5.8)	11 (7.1)	11 (7.1)	42 (27.1)	<0.001
Any respiratory hospitalization or death, *n* (%)	10 (6.5)	11 (7.1)	12 (7.7)	48 (31.0)	<0.001
Absolute decline in percent predicted DLco ≥15% or death, ^§^ *n* (%)	6 (6.3) ^||^	9 (9.0) ^¶^	9 (10.8) **	19 (28.4) ^††^	<0.001

* The Cochran–Armitage test for linear trend used quartile integers (1, 2, 3 and 4) as scores. Sensitivity analyses using median changes as scores for the quartiles did not result in meaningful differences. ^†^ Baseline assessments are defined as the last value obtained prior to first dose. ^‡^ For patients who died or discontinued prior to Month 12, the last available post-baseline value was used. ^§^ Post-baseline percent predicted DLco was only measured in CAPACITY. Quartiles were not redefined for this subset. ^||^ *n* = 95. ^¶^ *n* = 100. ** *n* = 83. ^††^ *n* = 67. 6MWD: 6-minute walking distance; DLco: diffusing capacity for carbon monoxide; FVC: forced vital capacity; IPF: idiopathic pulmonary fibrosis; NLR: neutrophil–lymphocyte ratio; Q: quartile; UCSD-SOBQ: University of California San Diego Shortness of Breath Questionnaire.

**Table 4:** All-cause mortality based on quartiles as defined by NLR and PLR changes from baseline to Month 12 in patients with IPF (pooled from the placebo groups of GIPF-001 and GIPF-007).

	Q1	Q2	Q3	Q4	Cochran–Armitage *p*-Value *
NLR, *n*	110	109	109	109	–
Changes from baseline ^†^ to Month 12, ^‡^ median (Q1, Q3)	−1.8 (−3.8, −1.2)	−0.2 (−0.5, −0.1)	0.5 (0.2, 0.8)	2.8 (1.6, 4.9)	–
All-cause mortality, *n* (%)	8 (7.3)	3 (2.8)	6 (5.5)	21 (19.3)	0.001
PLR, *n*	108	108	108	107	–
Changes from baseline ^†^ to Month 12, ^‡^ median (Q1, Q3)	−53.3 (−95.9, −34.0)	−10.6 (−16.4, −4.5)	14.9 (7.5, 22.2)	81.4 (56.9, 137.6)	–
All-cause mortality, *n* (%)	9 (8.3)	2 (1.9)	9 (8.3)	18 (16.8)	0.01

* The Cochran–Armitage test for linear trend used quartile integers (1, 2, 3 and 4) as scores. ^†^ Baseline assessments are defined as the last value obtained prior to first dose. ^‡^ For patients who died or discontinued prior to Month 12, the last available post-baseline value was used. IPF: idiopathic pulmonary fibrosis; NLR: neutrophil–lymphocyte ratio; PLR: platelet–lymphocyte ratio; Q: quartile.

## Data Availability

Qualified researchers may request access to individual patient level data through the clinical study data request platform (https://vivli.org (Accessed on 26 March 2021)). Further details on Roche’s criteria for eligible studies are available here (https://vivli.org/members/ourmembers (Accessed on 26 March 2021)). For further details on Roche’s Global Policy on the Sharing of Clinical Information and how to request access to related clinical study documents, see here (https://www.roche.com/research_and_development/who_we_are_how_we_work/clinical_trials/our_commitment_to_data_sharing.htm (Accessed on 26 March 2021)).

## Supplementary Materials

The following are available online at https://www.mdpi.com/article/10.3390/jcm10071427/s1, Table S1. Baseline demographic and clinical characteristics of patients included in ASCEND and CAPACITY (placebo and pirfenidone 2403 mg/day groups), by change from baseline to Month 12 in PLR. Table S2. Baseline demographic and clinical characteristics of patients included in ASCEND and CAPACITY (placebo and pirfenidone 2403 mg/day groups), by baseline NLR. Table S3. Baseline demographic and clinical characteristics of patients included in ASCEND and CAPACITY (placebo and pirfenidone 2403 mg/day groups), by baseline PLR. Table S4. *p*-values for Month 12 endpoints based on quartiles as defined by baseline NLR, baseline PLR, and NLR or PLR changes from baseline to Month 12 in patients with IPF. Table S5. Month 12 endpoints based on quartiles as defined by PLR changes from baseline to Month 12 in patients with IPF (pooled from the placebo groups of ASCEND and CAPACITY).
