# Supplementary material for: Changes in Neutrophil–Lymphocyte or Platelet–Lymphocyte Ratios and Their Associations with Clinical Outcomes in Idiopathic Pulmonary Fibrosis
Source: J Clin Med. 2021 Apr 1;10(7):1427. doi: 10.3390/jcm10071427 (PMC8037167; doi:10.3390/jcm10071427)
Supplement: Supplementary file 1 [file jcm-10-01427-s001.zip › NLRPLR Manuscript - Supplementary Materials/Nathan et al_NLR and PLR manuscript_Supplementary Materials.pdf]

Supplementary Materials

for

Changes in Neutrophil–Lymphocyte or Platelet–Lymphocyte Ratios and their Associations with Clinical Outcomes in Idiopathic Pulmonary Fibrosis

Steven D. Nathan, Jayesh Mehta, John Stauffer, Elizabeth Morgenthien, Ming Yang,

Susan L. Limb and Sangeeta Bhorade

Table S1. Baseline demographic and clinical characteristics of patients included in ASCEND and CAPACITY (placebo and pirfenidone 2403 mg/day groups), by change from baseline to Month 12 in PLR.

	**Placebo**				**Pirfenidone 2403 mg/day**			
	**Q1 (*n* = 154)**	**Q2 (*n* = 153)**	**Q3 (*n* = 154)**	**Q4 (*n* = 153)**	**Q1 (*n* = 154)**	**Q2 (*n* = 154)**	**Q3 (*n* = 154)**	**Q4 (*n* = 154)**
Male sex, *n* (%)	119 (77.3)	109 (71.2)	126 (81.8)	103 (67.3)	115 (74.7)	116 (75.3)	111 (72.1)	117 (76.0)
Age, yr, mean (SD)	67.3 (7.3)	65.6 (7.6)	67.9 (7.4)	67.8 (7.6)	66.3 (8.2)	67.7 (7.5)	66.5 (7.5)	68.3 (7.0)
Percent predicted FVC, mean (SD)	71.2 (13.1)	74.3 (13.7)	71.5 (13.3)	70.9 (13.9)	72.1 (12.7)	71.8 (12.8)	71.7 (13.9)	70.8 (13.2)
Percent predicted DLco, mean (SD)	44.8 (9.7)	47.1 (9.2)*	43.7 (9.1)	46.9 (15.1)*	45.7 (9.4)	45.2 (10.0)	46.4 (11.1)	45.0 (10.4)
Haemoglobin count, g/l, median (Q1, Q3)	142.0 (131.0, 150.0)	142.0 (134.0, 152.0)	142.0 (136.0, 151.0)	141.0 (132.0, 150.0)	141.0 (132.0, 151.0)	142.0 (134.0, 150.0)	145.0 (134.0, 152.0)	142.0 (135.0, 149.0)
Haematocrit count, median (Q1, Q3)	0.43 (0.40, 0.45)	0.42 (0.40, 0.45)	0.43 (0.40, 0.46)	0.42 (0.39, 0.45)	0.42 (0.39, 0.45)	0.42 (0.40, 0.45)	0.43 (0.40, 0.45)	0.42 (0.40, 0.45)
Platelet count, GI/l, median (Q1, Q3)	251.0 (217.0, 299.0)	227.0 (189.0, 264.0)	229.5 (196.0, 270.0)	252.0 (213.0, 298.0)	250.5 (211.0, 298.0)	234.5 (197.0, 281.0)	230.0 (204.0, 262.0)	238.5 (201.0, 280.0)

White blood cell count, GI/l, median (Q1, Q3)	7.7 (6.6, 9.2)	7.8 (6.8, 8.8)	8.2 (7.1, 9.6)	8.0 (7.1, 9.4)	7.9 (6.4, 9.2)	8.1 (7.0, 9.2)	7.8 (6.9, 9.3)	7.8 (6.6, 8.8)
Neutrophil count, GI/l, median (Q1, Q3)	5.2 (4.3, 6.4)	4.7 (4.0, 5.6)	5.1 (4.2, 6.0)	5.0 (4.2, 6.2)	5.1 (3.9, 6.3)	4.9 (4.2, 6.0)	4.8 (3.8, 5.8)	4.8 (3.9, 6.0)
Lymphocyte count, GI/l, median (Q1, Q3)	1.7 (1.4, 2.0)	2.1 (1.8, 2.7)	2.2 (1.8, 2.6)	2.2 (1.7, 2.7)	1.8 (1.4, 2.2)	2.2 (1.8, 2.6)	2.1 (1.8, 2.6)	2.0 (1.5, 2.5)
Monocyte count, GI/l, median (Q1, Q3)	0.45 (0.35, 0.57)	0.45 (0.38, 0.55)	0.50 (0.39, 0.60)	0.49 (0.37, 0.59)	0.45 (0.38, 0.56)	0.48 (0.40, 0.58)	0.49 (0.38, 0.58)	0.47 (0.40, 0.56)
Eosinophil count, GI/l, median (Q1, Q3)	0.21 (0.15, 0.34)	0.19 (0.12, 0.28)	0.24 (0.15, 0.36)	0.25 (0.16, 0.33)	0.20 (0.13, 0.29)	0.21 (0.13, 0.31)	0.25 (0.17, 0.36)	0.22 (0.14, 0.34)
Basophil count, GI/l, median (Q1, Q3)	0.05 (0.03, 0.07)	0.05 (0.04, 0.06)	0.06 (0.04, 0.07)	0.05 (0.03, 0.07)	0.05 (0.03, 0.07)	0.05 (0.04, 0.07)	0.05 (0.04, 0.07)	0.05 (0.04, 0.06)
NLR, median (Q1, Q3)	3.1 (2.5, 4.0)	2.2 (1.7, 3.0)	2.3 (1.8, 3.0)	2.4 (1.7, 3.2)	2.8 (2.1, 3.9)	2.3 (1.7, 3.0)	2.2 (1.7, 2.8)	2.5 (1.7, 3.4)
PLR, median (Q1, Q3)	150.8 (129.7, 180.4)	102.2 (84.4, 128.1)	106.7 (83.2, 132.3)	116.1 (95.9, 148.8)	139.5 (112.1, 174.2)	107.6 (89.1, 137.2)	108.8 (86.0, 132.0)	123.8 (94.2, 151.5)

*n = 152.

DLco: diffusing capacity for carbon monoxide; FVC: forced vital capacity; GI: 10^9 cells; NLR: neutrophil–lymphocyte ratio; Q: quartile;

PLR: platelet–lymphocyte ratio; SD: standard deviation.

Table S2. Baseline demographic and clinical characteristics of patients included in ASCEND and CAPACITY (placebo and pirfenidone 2403 mg/day groups), by baseline NLR.

	**Placebo**				**Pirfenidone 2403 mg/day**			
	**Q1 (*n* = 156)**	**Q2 (*n* = 156)**	**Q3 (*n* = 156)**	**Q4 (*n* = 156)**	**Q1 (*n* = 156)**	**Q2 (*n* = 156)**	**Q3 (*n* = 157)**	**Q4 (*n* = 154)**
Male sex, *n* (%)	102 (65.4)	117 (75.0)	116 (74.4)	130 (83.3)	107 (68.6)	110 (70.5)	119 (75.8)	127 (82.5)
Age, yr, mean (SD)	66.3 (7.6)	67.0 (7.6)	67.0 (7.6)	68.2 (7.3)	65.7 (7.9)	67.3 (7.5)	67.7 (7.7)	68.2 (6.9)
Percent predicted FVC, mean (SD)	73.3 (14.0)	72.6 (14.4)	72.1 (12.8)	70.0 (13.1)	71.7 (12.4)	72.3 (13.2)	72.6 (13.9)	69.8 (13.1)
Percent predicted DLco, mean (SD)	47.3 (10.3)	46.4 (13.7)*	45.1 (10.0)*	43.6 (9.7)	47.4 (10.6)	46.1 (9.7)	45.5 (10.9)	43.3 (9.1)
Haemoglobin count, g/l, median (Q1, Q3)	140.5 (133.5, 151.0)	142.0 (132.5, 149.5)	142.0 (134.5, 151.0)	142.5 (132.0, 152.0)	142.0 (133.0, 150.0)	142.0 (133.0, 150.0)	143.0 (135.0, 152.0)	141.0 (133.0, 150.0)
Haematocrit count, median (Q1, Q3)	0.42 (0.40, 0.46)	0.42 (0.40, 0.45)	0.42 (0.40, 0.45)	0.42 (0.39, 0.45)	0.42 (0.40, 0.45)	0.42 (0.39, 0.45)	0.43 (0.40, 0.45)	0.43 (0.40, 0.45)
Platelet count, GI/l, median (Q1, Q3)	232.0 (196.0, 276.0)	243.0 (212.0, 297.0)	247.0 (201.5, 287.5)	238.5 (202.5, 284.0)	234.5 (202.5, 264.5)	241.0 (197.0, 283.0)	235.0 (204.0, 282.0)	243.0 (206.0, 290.0)
White blood cell count, GI/l, median (Q1, Q3)	7.4 (6.2, 8.5)	7.7 (6.8, 8.9)	7.9 (7.1, 9.0)	8.8 (7.6, 10.2)	7.5 (6.1, 8.5)	7.5 (6.5, 8.8)	8.1 (6.9, 9.1)	8.6 (7.3, 10.0)

Neutrophil count, GI/l, median (Q1, Q3)	4.0 (3.3, 4.5)	4.8 (4.2, 5.5)	5.3 (4.8, 6.0)	6.3 (5.4, 7.6)	3.9 (3.1, 4.6)	4.5 (3.8, 5.2)	5.3 (4.6, 6.1)	6.2 (5.2, 7.4)
Lymphocyte count, GI/l, median (Q1, Q3)	2.6 (2.2, 3.1)	2.2 (1.9, 2.5)	1.8 (1.6, 2.2)	1.5 (1.2, 1.8)	2.8 (2.2, 3.2)	2.1 (1.8, 2.5)	1.9 (1.6, 2.2)	1.5 (1.2, 1.8)
Monocyte count, GI/l, median (Q1, Q3)	0.46 (0.36, 0.57)	0.47 (0.38, 0.56)	0.46 (0.35, 0.57)	0.49 (0.39, 0.62)	0.45 (0.37, 0.55)	0.48 (0.40, 0.58)	0.48 (0.39, 0.57)	0.51 (0.39, 0.62)
Eosinophil count, GI/l, median (Q1, Q3)	0.23 (0.15, 0.33)	0.20 (0.13, 0.30)	0.24 (0.15, 0.35)	0.21 (0.13, 0.33)	0.22 (0.13, 0.32)	0.24 (0.16, 0.36)	0.21 (0.14, 0.31)	0.21 (0.12, 0.33)
Basophil count, GI/l, median (Q1, Q3)	0.05 (0.03, 0.07)	0.05 (0.04, 0.07)	0.05 (0.04, 0.07)	0.05 (0.03, 0.06)	0.05 (0.03, 0.06)	0.05 (0.04, 0.08)	0.05 (0.04, 0.07)	0.05 (0.04, 0.07)
NLR, median (Q1, Q3)	1.5 (1.3, 1.7)	2.2 (2.0, 2.4)	2.9 (2.7, 3.1)	4.1 (3.6, 4.9)	1.5 (1.2, 1.6)	2.1 (2.0, 2.3)	2.8 (2.6, 3.0)	4.0 (3.6, 4.8)
PLR, median (Q1, Q3)	90.8 (71.2, 107.7)[†]	110.1 (93.3, 135.8)[*]	130.9 (106.5, 153.7)	162.8 (133.3, 195.0)[‡]	88.0 (70.9, 103.9)[*]	113.7 (92.9, 131.9)[*]	126.1 (107.8, 145.6)[§]	164.9 (136.1, 202.7)

[*] *n* = 155.

[†] *n* = 154.

[‡] *n* = 153.

[§] *n* = 156.

DLco: diffusing capacity for carbon monoxide; FVC: forced vital capacity; GI: 10^9 cells; NLR: neutrophil–lymphocyte ratio; Q: quartile; PLR: platelet–lymphocyte ratio; SD: standard deviation.

Table S3. Baseline demographic and clinical characteristics of patients included in ASCEND and CAPACITY (placebo and pirfenidone 2403 mg/day groups), by baseline PLR.

	**Placebo**				**Pirfenidone 2403 mg/day**			
	**Q1 (*n* = 155)**	**Q2 (*n* = 154)**	**Q3 (*n* = 155)**	**Q4 (*n* = 154)**	**Q1 (*n* = 155)**	**Q2 (*n* = 155)**	**Q3 (*n* = 155)**	**Q4 (*n* = 155)**
Male sex, *n* (%)	121 (78.1)	110 (71.4)	117 (75.5)	113 (73.4)	121 (78.1)	119 (76.8)	109 (70.3)	112 (72.3)
Age, yr, mean (SD)	66.5 (8.1)	67.2 (7.3)	67.4 (7.5)	67.7 (7.3)	67.1 (8.0)	66.2 (7.5)	67.9 (7.1)	67.6 (7.6)
Percent predicted FVC, mean (SD)	71.8 (13.3)	74.6 (14.0)	70.9 (13.8)	70.6 (12.8)	70.4 (11.4)	72.0 (13.3)	73.7 (14.4)	70.0 (13.0)
Percent predicted DLco, mean (SD)	45.7 (9.7)	46.7 (10.2)	45.0 (14.0)[*]	45.0 (10.1)[†]	44.3 (9.5)	46.6 (11.0)	46.4 (10.1)	44.9 (10.0)
Haemoglobin count, g/l, median (Q1, Q3)	144.0 (135.0, 153.0)	141.0 (136.0, 149.0)	140.0 (132.0, 151.0)	141.5 (129.0, 149.0)	145.0 (137.0, 152.0)	143.0 (133.0, 153.0)	142.0 (133.0, 150.0)	140.0 (131.0, 147.0)
Haematocrit count, median (Q1, Q3)	0.43 (0.41, 0.47)	0.42 (0.40, 0.45)	0.42 (0.40, 0.45)	0.42 (0.39, 0.45)	0.43 (0.40, 0.45)	0.43 (0.40, 0.46)	0.43 (0.40, 0.45)	0.42 (0.39, 0.44)
Platelet count, GI/l, median (Q1, Q3)	197.0 (176.0, 239.0)	235.0 (205.0, 266.0)	252.0 (221.0, 296.0)	275.5 (239.0, 322.0)	206.0 (178.0, 246.0)	229.0 (200.0, 258.0)	248.0 (213.0, 288.0)	281.0 (241.0, 330.0)

White blood cell count, GI/l, median (Q1, Q3)	8.3 (7.1, 9.6)	7.8 (7.0, 9.0)	7.8 (6.9, 9.2)	7.8 (6.5, 8.9)	8.3 (7.3, 9.7)	7.9 (6.6, 9.1)	7.6 (6.4, 8.8)	7.8 (6.5, 8.9)
Neutrophil count, GI/l, median (Q1, Q3)	4.8 (4.0, 5.8)	4.8 (4.1, 5.8)	5.2 (4.4, 6.1)	5.3 (4.3, 6.3)	4.7 (3.7, 5.8)	4.8 (3.9, 5.9)	5.0 (4.0, 6.0)	5.2 (4.3, 6.4)
Lymphocyte count, GI/l, median (Q1, Q3)	2.7 (2.3, 3.1)	2.2 (1.9, 2.5)	1.9 (1.7, 2.1)	1.5 (1.3, 1.7)	2.9 (2.2, 3.2)	2.1 (1.9, 2.4)	1.9 (1.6, 2.2)	1.5 (1.2, 1.8)
Monocyte count, GI/l, median (Q1, Q3)	0.51 (0.40, 0.61)	0.46 (0.36, 0.56)	0.46 (0.36, 0.57)	0.45 (0.35, 0.55)	0.48 (0.40, 0.58)	0.47 (0.38, 0.57)	0.47 (0.37, 0.57)	0.48 (0.39, 0.58)
Eosinophil count, GI/l, median (Q1, Q3)	0.22 (0.13, 0.31)	0.21 (0.13, 0.31)	0.21 (0.15, 0.35)	0.22 (0.16, 0.34)	0.24 (0.14, 0.34)	0.21 (0.14, 0.36)	0.21 (0.13, 0.30)	0.22 (0.14, 0.36)
Basophil count, GI/l, median (Q1, Q3)	0.06 (0.04, 0.08)	0.05 (0.04, 0.07)	0.05 (0.04, 0.07)	0.05 (0.03, 0.06)	0.05 (0.03, 0.07)	0.05 (0.04, 0.07)	0.05 (0.04, 0.07)	0.05 (0.04, 0.07)
NLR, median (Q1, Q3)	1.7 (1.4, 2.3)	2.2 (1.8, 2.8)	2.8 (2.2, 3.3)	3.5 (2.9, 4.6)	1.7 (1.3, 2.2)	2.2 (1.7, 2.7)	2.6 (2.2, 3.3)	3.6 (2.8, 4.5)
PLR, median (Q1, Q3)	79.2 (66.6, 86.8)	105.2 (100.8, 111.9)	136.9 (128.1, 144.2)	180.3 (166.3, 212.9)	78.7 (69.0, 86.6)	105.3 (100.0, 113.6)	131.9 (125.6, 140.1)	181.9 (161.6, 208.6)

$^{*}n = 154$.

$^{\dagger}n = 153$.

DLco: diffusing capacity for carbon monoxide; FVC: forced vital capacity; GI: 10^9 cells; NLR: neutrophil–lymphocyte ratio; Q: quartile; PLR: platelet–lymphocyte ratio; SD: standard deviation.

Table S4. *P* values for Month 12 endpoints based on quartiles as defined by baseline NLR, baseline PLR and NLR or PLR changes from baseline to Month 12 in patients with IPF.

	Baseline NLR*		Baseline PLR*		NLR or PLR Changes from Baseline to 12 Months*	
	Placebo Group	Pirfenidone 2403 mg/day	Placebo Group	Pirfenidone 2403 mg/day	NLR Changes, Pirfenidone 2403 mg/day	PLR Changes, Pirfenidone 2403 mg/day
All-cause mortality	0.03	0.18	0.83	0.92	0.28	0.69
Absolute decline in percent predicted FVC ≥10% or death	0.08	0.005	0.37	0.29	0.04	0.61
Absolute decline in 6MWD ≥50 m or death	0.01	0.001	0.04	0.36	0.38	0.40
Worsening in UCSD-SOBQ score ≥20 points or death	0.42	0.02	0.64	0.87	0.47	0.58
Any respiratory hospitalisation	0.25	0.77	0.58	0.19	<0.001	0.07

Any respiratory hospitalisation or death	0.15	0.95	0.75	0.33	0.001	0.10
Absolute decline in percent predicted DLco ≥15% or death[†]	0.32	0.49	0.77	0.16	0.26	0.43

[*]Data analysed by Cochran–Armitage test for linear trend, which used quartile integers (1, 2, 3 and 4) as scores.

[†]Post-baseline percent predicted DLco was only measured in CAPACITY. Quartiles were not redefined for this subset.

6MWD: 6-minute walk distance; DLco: diffusing capacity for carbon monoxide; FVC: forced vital capacity; IPF: idiopathic pulmonary fibrosis; NLR: neutrophil–lymphocyte ratio; PLR: platelet–lymphocyte ratio; UCSD-SOBQ: University of California San Diego Shortness of Breath Questionnaire.

Table S5. Month 12 endpoints based on quartiles as defined by PLR changes from baseline to Month 12 in patients with IPF (pooled from the placebo groups of ASCEND and CAPACITY).

	**Q1 (*n* = 154)**	**Q2 (*n* = 153)**	**Q3 (*n* = 154)**	**Q4 (*n* = 153)**	**Cochran–Armitage *P* Value***
PLR changes from baseline† to Month 12,‡ median (Q1, Q3)	−35.7 (−48.0, −24.4)	−6.1 (−10.9, −1.7)	12.2 (7.6, 18.0)	49.0 (37.1, 70.5)	–
Platelets percent change from baseline to Month 12, median (Q1, Q3)	−4.6 (−16.4, 3.2)	−2.8 (−10.3, 6.4)	4.2 (−4.3, 15.1)	10.1 (−1.9, 22.1)	–
Lymphocytes percent change from baseline to Month 12, median (Q1, Q3)	27.3 (9.7, 43.4)	3.7 (−6.1, 13.8)	−7.9 (−14.2, 3.5)	−22.9 (−35.6, −15.1)	–
Absolute decline in percent predicted FVC from baseline to Month 12, median (Q1, Q3)	−4.4 (−9.7, −0.9)	−3.9 (−8.2, −0.7)	−5.0 (−11.2, −1.5)	−7.6 (−13.8, −3.8)	–
All-cause mortality, *n* (%)	6 (3.9)	7 (4.6)	11 (7.1)	16 (10.5)	0.01
Absolute decline in percent predicted FVC ≥10% or death, *n* (%)	32 (20.8)	23 (15.0)	46 (29.9)	60 (39.2)	<0.001
Absolute decline in 6MWD ≥50 m or death, *n* (%)	50 (32.5)	39 (25.5)	54 (35.1)	66 (43.1)	0.02
Worsening in UCSD-SOBQ score ≥20 points or death, *n* (%)	42 (27.3)	33 (21.6)	45 (29.2)	71 (46.4)	<0.001

Any respiratory hospitalisation, *n* (%)	12 (7.8)	14 (9.2)	16 (10.4)	30 (19.6)	0.002
Any respiratory hospitalisation or death, *n* (%)	13 (8.4)	14 (9.2)	18 (11.7)	35 (22.9)	<0.001
Absolute decline in percent predicted DLco ≥15% or death,[§] *n* (%)	11 (12.0)[‖]	6 (5.9)[¶]	9 (10.8)[**]	17 (27.0)[††]	0.009

[*]The Cochran–Armitage test for linear trend used quartile integers (1, 2, 3 and 4) as scores. Sensitivity analyses using median changes as scores for the quartiles did not result in meaningful differences.

[†]Baseline assessments are defined as the last value obtained prior to first dose.

[‡]For patients who died or discontinued prior to Month 12, the last available post-baseline value was used.

[§]Post-baseline percent predicted DLco was only measured in CAPACITY. Quartiles were not redefined for this subset.

[‖]*n* = 92.

[¶]*n* = 102.

[**]*n* = 83.

[††]*n* = 63.

6MWD: 6-minute walk distance; DLco: diffusing capacity for carbon monoxide; FVC: forced vital capacity; IPF: idiopathic pulmonary fibrosis; PLR: platelet–lymphocyte ratio; Q: quartile; UCSD-SOBQ: University of California San Diego Shortness of Breath Questionnaire.
