# Supplementary material for: Changes in Neutrophil–Lymphocyte or Platelet–Lymphocyte Ratios and Their Associations with Clinical Outcomes in Idiopathic Pulmonary Fibrosis
Source: J Clin Med. 2021 Apr 1;10(7):1427. doi: 10.3390/jcm10071427 (PMC8037167; doi:10.3390/jcm10071427)
Supplement: Supplementary file 1 [file jcm-10-01427-s001.zip › NLRPLR Manuscript - Supplementary Materials/Nathan et al_NLR and PLR manuscript_Supplementary Materials_tracked changes.docx]

**Supplementary Materials**

*for*

**Changes in Neutrophil–Lymphocyte or Platelet–Lymphocyte Ratios and their Associations with Clinical Outcomes in Idiopathic Pulmonary Fibrosis**

Steven D. Nathan, Jayesh Mehta, John Stauffer, Elizabeth Morgenthien, Ming Yang, Susan L. Limb and Sangeeta Bhorade

**Table S1.** Baseline demographic and clinical characteristics of patients included in ASCEND and CAPACITY (placebo and pirfenidone 2403 mg/day groups), by change from baseline to Month 12 in PLR.

|  | **Placebo** | | | | **Pirfenidone 2403 mg/day** | | | |
| --- | --- | --- | --- | --- | --- | --- | --- | --- |
|  | **Q1**  **(*n* = 154)** | **Q2**  **(*n* = 153)** | **Q3**  **(*n* = 154)** | **Q4**  **(*n* = 153)** | **Q1**  **(*n* = 154)** | **Q2**  **(*n* = 154)** | **Q3**  **(*n* = 154)** | **Q4**  **(*n* = 154)** |
| Male sex, *n* (%) | 119 (77.3) | 109 (71.2) | 126 (81.8) | 103 (67.3) | 115 (74.7) | 116 (75.3) | 111 (72.1) | 117 (76.0) |
| Age, yr, mean (SD) | 67.3 (7.3) | 65.6 (7.6) | 67.9 (7.4) | 67.8 (7.6) | 66.3 (8.2) | 67.7 (7.5) | 66.5 (7.5) | 68.3 (7.0) |
| Percent predicted FVC, mean (SD) | 71.2 (13.1) | 74.3 (13.7) | 71.5 (13.3) | 70.9 (13.9) | 72.1 (12.7) | 71.8 (12.8) | 71.7 (13.9) | 70.8 (13.2) |
| Percent predicted DLco, mean (SD) | 44.8 (9.7) | 47.1 (9.2)^*^ | 43.7 (9.1) | 46.9 (15.1)^*^ | 45.7 (9.4) | 45.2 (10.0) | 46.4 (11.1) | 45.0 (10.4) |
| Haemoglobin count, g/l, median (Q1, Q3) | 142.0 (131.0, 150.0) | 142.0 (134.0, 152.0) | 142.0 (136.0, 151.0) | 141.0 (132.0, 150.0) | 141.0 (132.0, 151.0) | 142.0 (134.0, 150.0) | 145.0 (134.0, 152.0) | 142.0 (135.0, 149.0) |
| Haematocrit count, median (Q1, Q3) | 0.43 (0.40, 0.45) | 0.42 (0.40, 0.45) | 0.43 (0.40, 0.46) | 0.42 (0.39, 0.45) | 0.42 (0.39, 0.45) | 0.42 (0.40, 0.45) | 0.43 (0.40, 0.45) | 0.42 (0.40, 0.45) |
| Platelet count, GI/l, median (Q1, Q3) | 251.0 (217.0, 299.0) | 227.0 (189.0, 264.0) | 229.5 (196.0, 270.0) | 252.0 (213.0, 298.0) | 250.5 (211.0, 298.0) | 234.5 (197.0, 281.0) | 230.0 (204.0, 262.0) | 238.5 (201.0, 280.0) |
| White blood cell count, GI/l, median (Q1, Q3) | 7.7 (6.6, 9.2) | 7.8 (6.8, 8.8) | 8.2 (7.1, 9.6) | 8.0 (7.1, 9.4) | 7.9 (6.4, 9.2) | 8.1 (7.0, 9.2) | 7.8 (6.9, 9.3) | 7.8 (6.6, 8.8) |
| Neutrophil count, GI/l, median (Q1, Q3) | 5.2 (4.3, 6.4) | 4.7 (4.0, 5.6) | 5.1 (4.2, 6.0) | 5.0 (4.2, 6.2) | 5.1 (3.9, 6.3) | 4.9 (4.2, 6.0) | 4.8 (3.8, 5.8) | 4.8 (3.9, 6.0) |
| Lymphocyte count, GI/l, median (Q1, Q3) | 1.7 (1.4, 2.0) | 2.1 (1.8, 2.7) | 2.2 (1.8, 2.6) | 2.2 (1.7, 2.7) | 1.8 (1.4, 2.2) | 2.2 (1.8, 2.6) | 2.1 (1.8, 2.6) | 2.0 (1.5, 2.5) |
| Monocyte count, GI/l, median (Q1, Q3) | 0.45 (0.35, 0.57) | 0.45 (0.38, 0.55) | 0.50 (0.39, 0.60) | 0.49 (0.37, 0.59) | 0.45 (0.38, 0.56) | 0.48 (0.40, 0.58) | 0.49 (0.38, 0.58) | 0.47 (0.40, 0.56) |
| Eosinophil count, GI/l, median (Q1, Q3) | 0.21 (0.15, 0.34) | 0.19 (0.12, 0.28) | 0.24 (0.15, 0.36) | 0.25 (0.16, 0.33) | 0.20 (0.13, 0.29) | 0.21 (0.13, 0.31) | 0.25 (0.17, 0.36) | 0.22 (0.14, 0.34) |
| Basophil count, GI/l, median (Q1, Q3) | 0.05 (0.03, 0.07) | 0.05 (0.04, 0.06) | 0.06 (0.04, 0.07) | 0.05 (0.03, 0.07) | 0.05 (0.03, 0.07) | 0.05 (0.04, 0.07) | 0.05 (0.04, 0.07) | 0.05 (0.04, 0.06) |
| NLR, median (Q1, Q3) | 3.1 (2.5, 4.0) | 2.2 (1.7, 3.0) | 2.3 (1.8, 3.0) | 2.4 (1.7, 3.2) | 2.8 (2.1, 3.9) | 2.3 (1.7, 3.0) | 2.2 (1.7, 2.8) | 2.5 (1.7, 3.4) |
| PLR, median (Q1, Q3) | 150.8 (129.7, 180.4) | 102.2 (84.4, 128.1) | 106.7 (83.2, 132.3) | 116.1 (95.9, 148.8) | 139.5 (112.1, 174.2) | 107.6 (89.1, 137.2) | 108.8 (86.0, 132.0) | 123.8 (94.2, 151.5) |

^*^*n* = 152.

DLco: diffusing capacity for carbon monoxide; FVC: forced vital capacity; GI: 10^9^ cells; NLR: neutrophil–lymphocyte ratio; Q: quartile; PLR: platelet–lymphocyte ratio; SD: standard deviation.

**Table S2.** Baseline demographic and clinical characteristics of patients included in ASCEND and CAPACITY (placebo and pirfenidone 2403 mg/day groups), by baseline NLR.

|  | **Placebo** | | | | **Pirfenidone 2403 mg/day** | | | |
| --- | --- | --- | --- | --- | --- | --- | --- | --- |
|  | **Q1**  **(*n* = 156)** | **Q2**  **(*n* = 156)** | **Q3**  **(*n* = 156)** | **Q4**  **(*n* = 156)** | **Q1**  **(*n* = 156)** | **Q2**  **(*n* = 156)** | **Q3**  **(*n* = 157)** | **Q4**  **(*n* = 154)** |
| Male sex, *n* (%) | 102 (65.4) | 117 (75.0) | 116 (74.4) | 130 (83.3) | 107 (68.6) | 110 (70.5) | 119 (75.8) | 127 (82.5) |
| Age, yr, mean (SD) | 66.3 (7.6) | 67.0 (7.6) | 67.0 (7.6) | 68.2 (7.3) | 65.7 (7.9) | 67.3 (7.5) | 67.7 (7.7) | 68.2 (6.9) |
| Percent predicted FVC, mean (SD) | 73.3 (14.0) | 72.6 (14.4) | 72.1 (12.8) | 70.0 (13.1) | 71.7 (12.4) | 72.3 (13.2) | 72.6 (13.9) | 69.8 (13.1) |
| Percent predicted DLco, mean (SD) | 47.3 (10.3) | 46.4 (13.7)^*^ | 45.1 (10.0)^*^ | 43.6 (9.7) | 47.4 (10.6) | 46.1 (9.7) | 45.5 (10.9) | 43.3 (9.1) |
| Haemoglobin count, g/l, median (Q1, Q3) | 140.5 (133.5, 151.0) | 142.0 (132.5, 149.5) | 142.0 (134.5, 151.0) | 142.5 (132.0, 152.0) | 142.0 (133.0, 150.0) | 142.0 (133.0, 150.0) | 143.0 (135.0, 152.0) | 141.0 (133.0, 150.0) |
| Haematocrit count, median (Q1, Q3) | 0.42 (0.40, 0.46) | 0.42 (0.40, 0.45) | 0.42 (0.40, 0.45) | 0.42 (0.39, 0.45) | 0.42 (0.40, 0.45) | 0.42 (0.39, 0.45) | 0.43 (0.40, 0.45) | 0.43 (0.40, 0.45) |
| Platelet count, GI/l, median (Q1, Q3) | 232.0 (196.0, 276.0) | 243.0 (212.0, 297.0) | 247.0 (201.5, 287.5) | 238.5 (202.5, 284.0) | 234.5 (202.5, 264.5) | 241.0 (197.0, 283.0) | 235.0 (204.0, 282.0) | 243.0 (206.0, 290.0) |
| White blood cell count, GI/l, median (Q1, Q3) | 7.4 (6.2, 8.5) | 7.7 (6.8, 8.9) | 7.9 (7.1, 9.0) | 8.8 (7.6, 10.2) | 7.5 (6.1, 8.5) | 7.5 (6.5, 8.8) | 8.1 (6.9, 9.1) | 8.6 (7.3, 10.0) |
| Neutrophil count, GI/l, median (Q1, Q3) | 4.0 (3.3, 4.5) | 4.8 (4.2, 5.5) | 5.3 (4.8, 6.0) | 6.3 (5.4, 7.6) | 3.9 (3.1, 4.6) | 4.5 (3.8, 5.2) | 5.3 (4.6, 6.1) | 6.2 (5.2, 7.4) |
| Lymphocyte count, GI/l, median (Q1, Q3) | 2.6 (2.2, 3.1) | 2.2 (1.9, 2.5) | 1.8 (1.6, 2.2) | 1.5 (1.2, 1.8) | 2.8 (2.2, 3.2) | 2.1 (1.8, 2.5) | 1.9 (1.6, 2.2) | 1.5 (1.2, 1.8) |
| Monocyte count, GI/l, median (Q1, Q3) | 0.46 (0.36, 0.57) | 0.47 (0.38, 0.56) | 0.46 (0.35, 0.57) | 0.49 (0.39, 0.62) | 0.45 (0.37, 0.55) | 0.48 (0.40, 0.58) | 0.48 (0.39, 0.57) | 0.51 (0.39, 0.62) |
| Eosinophil count, GI/l, median (Q1, Q3) | 0.23 (0.15, 0.33) | 0.20 (0.13, 0.30) | 0.24 (0.15, 0.35) | 0.21 (0.13, 0.33) | 0.22 (0.13, 0.32) | 0.24 (0.16, 0.36) | 0.21 (0.14, 0.31) | 0.21 (0.12, 0.33) |
| Basophil count, GI/l, median (Q1, Q3) | 0.05 (0.03, 0.07) | 0.05 (0.04, 0.07) | 0.05 (0.04, 0.07) | 0.05 (0.03, 0.06) | 0.05 (0.03, 0.06) | 0.05 (0.04, 0.08) | 0.05 (0.04, 0.07) | 0.05 (0.04, 0.07) |
| NLR, median (Q1, Q3) | 1.5 (1.3, 1.7) | 2.2 (2.0, 2.4) | 2.9 (2.7, 3.1) | 4.1 (3.6, 4.9) | 1.5 (1.2, 1.6) | 2.1 (2.0, 2.3) | 2.8 (2.6, 3.0) | 4.0 (3.6, 4.8) |
| PLR, median (Q1, Q3) | 90.8 (71.2,  107.7)^†^ | 110.1 (93.3, 135.8)^*^ | 130.9 (106.5, 153.7) | 162.8 (133.3, 195.0)^‡^ | 88.0 (70.9,  103.9)^*^ | 113.7 (92.9, 131.9)^*^ | 126.1 (107.8, 145.6)^§^ | 164.9 (136.1, 202.7) |

^*^*n* = 155.

^†^*n* = 154.

^‡^*n* = 153.

^§^*n* = 156.

DLco: diffusing capacity for carbon monoxide; FVC: forced vital capacity; GI: 10^9^ cells; NLR: neutrophil–lymphocyte ratio; Q: quartile; PLR: platelet–lymphocyte ratio; SD: standard deviation.

**Table S3.** Baseline demographic and clinical characteristics of patients included in ASCEND and CAPACITY (placebo and pirfenidone 2403 mg/day groups), by baseline PLR.

|  | **Placebo** | | | | **Pirfenidone 2403 mg/day** | | | |
| --- | --- | --- | --- | --- | --- | --- | --- | --- |
|  | **Q1**  **(*n* = 155)** | **Q2**  **(*n* = 154)** | **Q3**  **(*n* = 155)** | **Q4**  **(*n* = 154)** | **Q1**  **(*n* = 155)** | **Q2**  **(*n* = 155)** | **Q3**  **(*n* = 155)** | **Q4**  **(*n* = 155)** |
| Male sex, *n* (%) | 121 (78.1) | 110 (71.4) | 117 (75.5) | 113 (73.4) | 121 (78.1) | 119 (76.8) | 109 (70.3) | 112 (72.3) |
| Age, yr, mean (SD) | 66.5 (8.1) | 67.2 (7.3) | 67.4 (7.5) | 67.7 (7.3) | 67.1 (8.0) | 66.2 (7.5) | 67.9 (7.1) | 67.6 (7.6) |
| Percent predicted FVC, mean (SD) | 71.8 (13.3) | 74.6 (14.0) | 70.9 (13.8) | 70.6 (12.8) | 70.4 (11.4) | 72.0 (13.3) | 73.7 (14.4) | 70.0 (13.0) |
| Percent predicted DLco, mean (SD) | 45.7 (9.7) | 46.7 (10.2) | 45.0 (14.0)^*^ | 45.0 (10.1)^†^ | 44.3 (9.5) | 46.6 (11.0) | 46.4 (10.1) | 44.9 (10.0) |
| Haemoglobin count, g/l, median (Q1, Q3) | 144.0 (135.0, 153.0) | 141.0 (136.0, 149.0) | 140.0 (132.0, 151.0) | 141.5 (129.0, 149.0) | 145.0 (137.0, 152.0) | 143.0 (133.0, 153.0) | 142.0 (133.0, 150.0) | 140.0 (131.0, 147.0) |
| Haematocrit count, median (Q1, Q3) | 0.43 (0.41, 0.47) | 0.42 (0.40, 0.45) | 0.42 (0.40, 0.45) | 0.42 (0.39, 0.45) | 0.43 (0.40, 0.45) | 0.43 (0.40, 0.46) | 0.43 (0.40, 0.45) | 0.42 (0.39, 0.44) |
| Platelet count, GI/l, median (Q1, Q3) | 197.0 (176.0, 239.0) | 235.0 (205.0, 266.0) | 252.0 (221.0, 296.0) | 275.5 (239.0, 322.0) | 206.0 (178.0, 246.0) | 229.0 (200.0, 258.0) | 248.0 (213.0, 288.0) | 281.0 (241.0, 330.0) |
| White blood cell count, GI/l, median (Q1, Q3) | 8.3 (7.1, 9.6) | 7.8 (7.0, 9.0) | 7.8 (6.9, 9.2) | 7.8 (6.5, 8.9) | 8.3 (7.3, 9.7) | 7.9 (6.6, 9.1) | 7.6 (6.4, 8.8) | 7.8 (6.5, 8.9) |
| Neutrophil count, GI/l, median (Q1, Q3) | 4.8 (4.0, 5.8) | 4.8 (4.1, 5.8) | 5.2 (4.4, 6.1) | 5.3 (4.3, 6.3) | 4.7 (3.7, 5.8) | 4.8 (3.9, 5.9) | 5.0 (4.0, 6.0) | 5.2 (4.3, 6.4) |
| Lymphocyte count, GI/l, median (Q1, Q3) | 2.7 (2.3, 3.1) | 2.2 (1.9, 2.5) | 1.9 (1.7, 2.1) | 1.5 (1.3, 1.7) | 2.9 (2.2, 3.2) | 2.1 (1.9, 2.4) | 1.9 (1.6, 2.2) | 1.5 (1.2, 1.8) |
| Monocyte count, GI/l, median (Q1, Q3) | 0.51 (0.40, 0.61) | 0.46 (0.36, 0.56) | 0.46 (0.36, 0.57) | 0.45 (0.35, 0.55) | 0.48 (0.40, 0.58) | 0.47 (0.38, 0.57) | 0.47 (0.37, 0.57) | 0.48 (0.39, 0.58) |
| Eosinophil count, GI/l, median (Q1, Q3) | 0.22 (0.13, 0.31) | 0.21 (0.13, 0.31) | 0.21 (0.15, 0.35) | 0.22 (0.16, 0.34) | 0.24 (0.14, 0.34) | 0.21 (0.14, 0.36) | 0.21 (0.13, 0.30) | 0.22 (0.14, 0.36) |
| Basophil count, GI/l, median (Q1, Q3) | 0.06 (0.04, 0.08) | 0.05 (0.04, 0.07) | 0.05 (0.04, 0.07) | 0.05 (0.03, 0.06) | 0.05 (0.03, 0.07) | 0.05 (0.04, 0.07) | 0.05 (0.04, 0.07) | 0.05 (0.04, 0.07) |
| NLR, median (Q1, Q3) | 1.7 (1.4, 2.3) | 2.2 (1.8, 2.8) | 2.8 (2.2, 3.3) | 3.5 (2.9, 4.6) | 1.7 (1.3, 2.2) | 2.2 (1.7, 2.7) | 2.6 (2.2, 3.3) | 3.6 (2.8, 4.5) |
| PLR, median (Q1, Q3) | 79.2 (66.6, 86.8) | 105.2 (100.8, 111.9) | 136.9 (128.1, 144.2) | 180.3 (166.3, 212.9) | 78.7 (69.0, 86.6) | 105.3 (100.0, 113.6) | 131.9 (125.6, 140.1) | 181.9 (161.6, 208.6) |

^*^*n* = 154.

^†^*n* = 153.

DLco: diffusing capacity for carbon monoxide; FVC: forced vital capacity; GI: 10^9^ cells; NLR: neutrophil–lymphocyte ratio; Q: quartile; PLR: platelet–lymphocyte ratio; SD: standard deviation.

**Table S4.** *P* values for Month 12 endpoints based on quartiles as defined by baseline NLR, baseline PLR and NLR or PLR changes from baseline to Month 12 in patients with IPF.

|  | **Baseline NLR^*^** | | **Baseline PLR^*^** | | **NLR or PLR Changes from Baseline to 12 Months^*^** | |
| --- | --- | --- | --- | --- | --- | --- |
|  | **Placebo Group** | **Pirfenidone 2403 mg/day** | **Placebo Group** | **Pirfenidone 2403 mg/day** | **NLR Changes, Pirfenidone 2403 mg/day** | **PLR Changes, Pirfenidone 2403 mg/day** |
| All-cause mortality | 0.03 | 0.18 | 0.83 | 0.92 | 0.28 | 0.69 |
| Absolute decline in percent predicted FVC ≥10% or death | 0.08 | 0.005 | 0.37 | 0.29 | 0.04 | 0.61 |
| Absolute decline in 6MWD ≥50 m or death | 0.01 | 0.001 | 0.04 | 0.36 | 0.38 | 0.40 |
| Worsening in UCSD-SOBQ score ≥20 points or death | 0.42 | 0.02 | 0.64 | 0.87 | 0.47 | 0.58 |
| Any respiratory hospitalisation | 0.25 | 0.77 | 0.58 | 0.19 | <0.001 | 0.07 |
| Any respiratory hospitalisation or death | 0.15 | 0.95 | 0.75 | 0.33 | 0.001 | 0.10 |
| Absolute decline in percent predicted DLco ≥15% or death^†^ | 0.32 | 0.49 | 0.77 | 0.16 | 0.26 | 0.43 |

^*^Data analysed by Cochran–Armitage test for linear trend, which used quartile integers (1, 2, 3 and 4) as scores.

^†^Post-baseline percent predicted DLco was only measured in CAPACITY. Quartiles were not redefined for this subset.

6MWD: 6-minute walk distance; DLco: diffusing capacity for carbon monoxide; FVC: forced vital capacity; IPF: idiopathic pulmonary fibrosis; NLR: neutrophil–lymphocyte ratio; PLR: platelet–lymphocyte ratio; UCSD-SOBQ: University of California San Diego Shortness of Breath Questionnaire.

**Table S5.** Month 12 endpoints based on quartiles as defined by PLR changes from baseline to Month 12 in patients with IPF (pooled from the placebo groups of ASCEND and CAPACITY).

|  | **Q1 (*n* = 154)** | **Q2 (*n* = 153)** | **Q3 (*n* = 154)** | **Q4 (*n* = 153)** | **Cochran–Armitage  *P* Value^*^** |
| --- | --- | --- | --- | --- | --- |
| PLR changes from baseline^†^ to Month 12,^‡^ median (Q1, Q3) | −35.7 (−48.0, −24.4) | −6.1 (−10.9, −1.7) | 12.2 (7.6, 18.0) | 49.0 (37.1, 70.5) | – |
| Platelets percent change from baseline to Month 12, median (Q1, Q3) | −4.6 (−16.4, 3.2) | −2.8 (−10.3, 6.4) | 4.2 (−4.3, 15.1) | 10.1 (−1.9, 22.1) | – |
| Lymphocytes percent change from baseline to Month 12, median (Q1, Q3) | 27.3 (9.7, 43.4) | 3.7 (−6.1, 13.8) | −7.9 (−14.2, 3.5) | −22.9 (−35.6, −15.1) | – |
| Absolute decline in percent predicted FVC from baseline to Month 12, median (Q1, Q3) | −4.4  (−9.7, −0.9) | −3.9  (−8.2, −0.7) | −5.0  (−11.2, −1.5) | −7.6  (−13.8, −3.8) | – |
| All-cause mortality, *n* (%) | 6 (3.9) | 7 (4.6) | 11 (7.1) | 16 (10.5) | 0.01 |
| Absolute decline in percent predicted FVC ≥10% or death, *n* (%) | 32 (20.8) | 23 (15.0) | 46 (29.9) | 60 (39.2) | <0.001 |
| Absolute decline in 6MWD ≥50 m or death, *n* (%) | 50 (32.5) | 39 (25.5) | 54 (35.1) | 66 (43.1) | 0.02 |
| Worsening in UCSD-SOBQ score ≥20 points or death, *n* (%) | 42 (27.3) | 33 (21.6) | 45 (29.2) | 71 (46.4) | <0.001 |
| Any respiratory hospitalisation, *n* (%) | 12 (7.8) | 14 (9.2) | 16 (10.4) | 30 (19.6) | 0.002 |
| Any respiratory hospitalisation or death, *n* (%) | 13 (8.4) | 14 (9.2) | 18 (11.7) | 35 (22.9) | <0.001 |
| Absolute decline in percent predicted DLco ≥15% or death,^§^ *n* (%) | 11 (12.0)^\|\|^ | 6 (5.9)^¶^ | 9 (10.8)^**^ | 17 (27.0)^††^ | 0.009 |

^*^The Cochran–Armitage test for linear trend used quartile integers (1, 2, 3 and 4) as scores. Sensitivity analyses using median changes as scores for the quartiles did not result in meaningful differences.

^†^Baseline assessments are defined as the last value obtained prior to first dose.

^‡^For patients who died or discontinued prior to Month 12, the last available post-baseline value was used.

^§^Post-baseline percent predicted DLco was only measured in CAPACITY. Quartiles were not redefined for this subset.

^||^*n* = 92.

^¶^*n* = 102.

^**^*n* = 83.

^††^*n* = 63.

6MWD: 6-minute walk distance; DLco: diffusing capacity for carbon monoxide; FVC: forced vital capacity; IPF: idiopathic pulmonary fibrosis; PLR: platelet–lymphocyte ratio; Q: quartile; UCSD-SOBQ: University of California San Diego Shortness of Breath Questionnaire.
